# Ethical challenges with the left ventricular assist device as a destination therapy

**DOI:** 10.1186/1747-5341-3-20

**Published:** 2008-08-11

**Authors:** Aaron G Rizzieri, Joseph L Verheijde, Mohamed Y Rady, Joan L McGregor

**Affiliations:** 1 Department of Philosophy, Arizona State University, 300 East University Drive, Tempe, Arizona, 85287, USA; 2 Department of Physical Medicine and Rehabilitation, Mayo Clinic Hospital, Mayo Clinic Arizona, 5777 East Mayo Boulevard, Phoenix, Arizona, 85054, USA; 3 Bioethics, Policy, and Law Program, Arizona State University, 300 East University Drive, Tempe, Arizona, 85287, USA; 4 Department of Critical Care Medicine, Mayo Clinic Hospital, Mayo Clinic Arizona, 5777 East Mayo Boulevard, Phoenix, Arizona, 85054, USA

## Abstract

The left ventricular assist device was originally designed to be surgically implanted as a bridge to transplantation for patients with chronic end-stage heart failure. On the basis of the REMATCH trial, the US Food and Drug Administration and the US Centers for Medicare & Medicaid Services approved permanent implantation of the left ventricular assist device as a destination therapy in Medicare beneficiaries who are not candidates for heart transplantation. The use of the left ventricular assist device as a destination therapy raises certain ethical challenges. Left ventricular assist devices can prolong the survival of average recipients compared with optimal medical management of chronic end-stage heart failure. However, the overall quality of life can be adversely affected in some recipients because of serious infections, neurologic complications, and device malfunction. Left ventricular assist devices alter end-of-life trajectories. The caregivers of recipients may experience significant burden (e.g., poor physical health, depression, anxiety, and posttraumatic stress disorder) from destination therapy with left ventricular assist devices. There are also social and financial ramifications for recipients and their families. We advocate early utilization of a palliative care approach and outline prerequisite conditions so that consenting for the use of a left ventricular assist device as a destination therapy is a well informed process. These conditions include: (1) direct participation of a multidisciplinary care team, including palliative care specialists, (2) a concise plan of care for anticipated device-related complications, (3) careful surveillance and counseling for caregiver burden, (4) advance-care planning for anticipated end-of-life trajectories and timing of device deactivation, and (5) a plan to address the long-term financial burden on patients, families, and caregivers.

Short-term mechanical circulatory devices (e.g. percutaneous cardiopulmonary bypass, percutaneous ventricular assist devices, etc.) can be initiated in emergency situations as a bridge to permanent implantation of ventricular assist devices in chronic end-stage heart failure. In the absence of first-person (patient) consent, presumed consent or surrogate consent should be used cautiously for the initiation of short-term mechanical circulatory devices in emergency situations as a bridge to permanent implantation of left ventricular assist devices. Future clinical studies of destination therapy with left ventricular assist devices should include measures of recipients' quality of end-of-life care and caregivers' burden.

## Introduction

In clinical practice, several types of mechanical circulatory devices are used for the temporary support of the left or right heart functions or both [1-6]. The left ventricular assist device (LVAD) is a surgically implanted mechanical circulatory device used for temporary support of left heart function as a bridge to recovery of native function or to cardiac transplantation [7-9]. However, it is becoming increasingly common for the LVAD technology to be also used as a permanent destination therapy (DT) for patients with chronic end-stage heart failure who are not candidates for transplantation [10]. Using LVAD technology as DT (LVAD-DT) is intended to prolong survival, improve quality of life (QOL) and enhance functional status of patients with chronic end-stage heart failure. The benefits attributed to LVAD-DT are substantiated by outcome data obtained from several clinical studies [11-14]. In this article, we re-examine survival data and common types of complications attributed to LVAD-DT from the same clinical studies in chronic end-stage heart failure.

The complications of LVAD-DT can adversely influence QOL for patients and caregivers (e.g., spouses, adult children or grand-children, significant others, and close friends) alike and alter end-of-life trajectory in chronic end-stage heart failure. Therefore, the use of LVAD-DT raises certain ethical challenges that ought to be adequately addressed in clinical practice. The ethical challenges pertain to the risks and benefits of such devices and their consequences for end-of-life trajectories and care. To address these ethical challenges, we describe prerequisite conditions in the consent process for LVAD-DT to fulfill the goals of patient-centered and optimal end-of-life care, including palliation in patients who may choose LVAD-DT as an alternate therapy option, in chronic end-stage heart failure.

## Device implantation and survival

**Figure 1:**
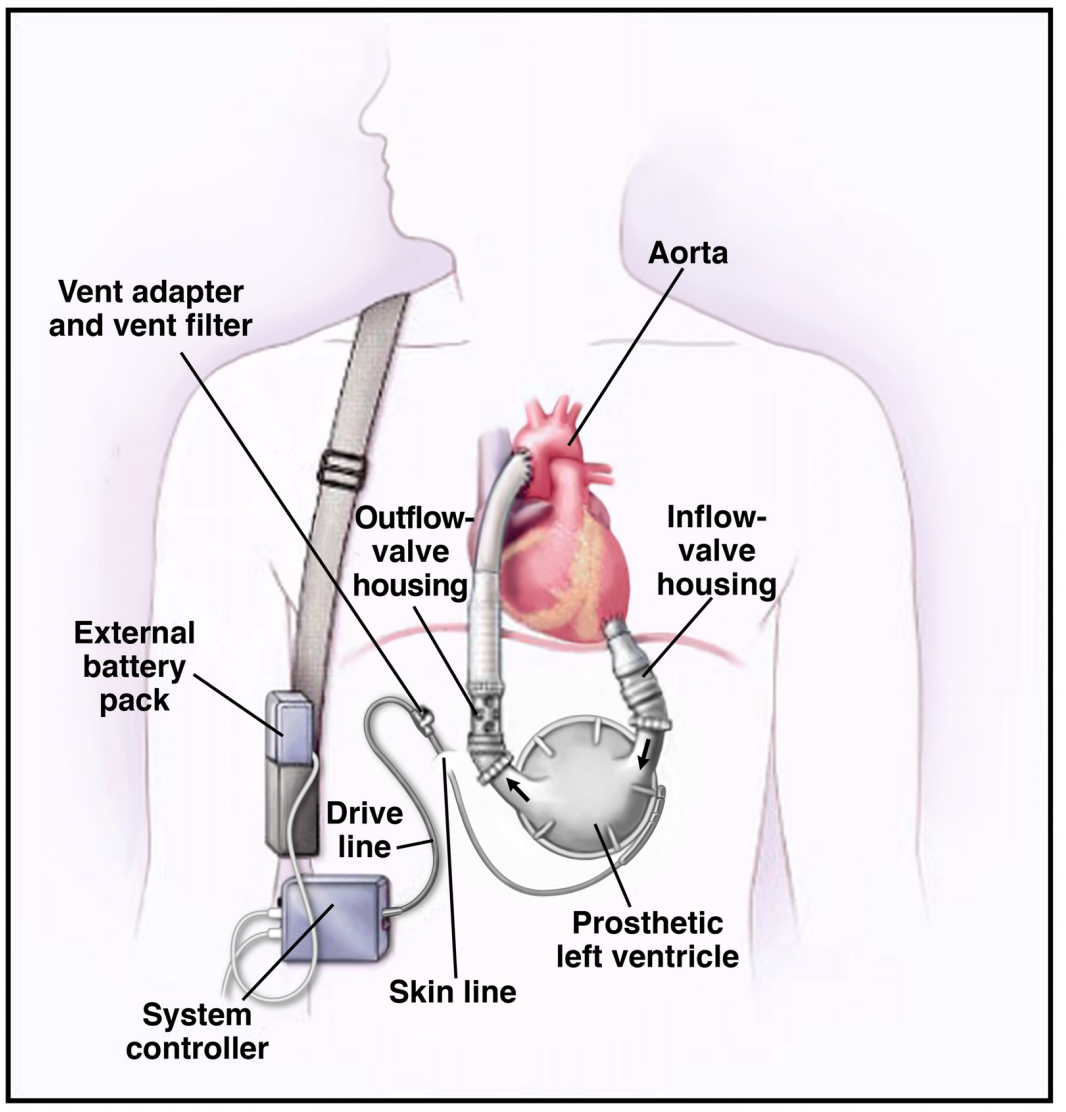
**The left ventricular assist device (LVAD)**. The LVAD from Thoratec Corporation (Pleasanton, California), the HeartMate LVAS (left ventricular assist system) XVE, helps the left ventricle of the heart pump blood throughout the body. A median sternotomy and cardiopulmonary bypass are required for access and implantation of the LVAD. The LVAD is implanted below the heart within the abdominal wall or peritoneal cavity. The LVAD is attached in parallel with the cardiovascular system. This leaves the heart connected to the circulatory system but provides the energy needed to propel blood throughout the body. The inflow cannula is anastomosed to the tip of the left ventricle so that blood is channeled into the device. An external control system triggers blood from the natural heart to fill the pump. A small motor drives the pump through an external battery-powered control unit. A pusher plate forces a flexible polyurethane diaphragm upward and pressurizes the blood chamber. This motion propels blood through an outflow conduit and a graft that is attached to the ascending aorta. The ascending aorta is the main artery supplying oxygen-rich blood throughout the body. Valves located on either side of the pumping chamber of the device keep blood flowing in one direction only. For more information, see Thoratec Web site[1]. (From . [Used with permission.]). See Supplemental file for Uniform Resource Locator (URL) links for videos on implantation procedures of ventricular assist devices.

LVAD implantation involves open heart surgery (Figure 1, and additional file 1: videos of HeartMate XVE and Thoratec ventricular assist devices implantation procedures). Surgery to implant the LVAD has a hospital (30- to 90-day) mortality of 14% to 27%, depending on the recipient's age and preexisting disease and comorbid conditions [11-13,15,16]. In a prospective, randomized, controlled trial (Randomized Evaluation of Mechanical Assistance for the Treatment of Congestive Heart Failure [REMATCH]) conducted between May 1998 and July 2001, 129 patients with chronic end-stage heart failure who were ineligible for cardiac transplantation were randomly assigned to receive LVAD-DT (n = 68) or optimal medical management (OMM) (n = 61) [11]. In this trial, the HeartMate XVE (Thoratec Corporation, Pleasanton, California) was implanted as LVAD-DT (Figure 1) [1]. The LVAD-DT group had superior survival over OMM at 1 year follow-up (52% vs 25%, *P *= 0.002) and at 2-year (23% vs 8%, *P *= 0.09). However, the preponderance of male patients enrolled in the REMATCH trial (82% and 78% male patients treated with OMM and LVAD-DT, respectively) made it difficult to generalize the survival benefits to female patients [11]. In spite of this limitation of the REMATCH trial, the US Food and Drug Administration and the US Centers for Medicare & Medicaid Services approved LVAD-DT in 2003 for men and women Medicare beneficiaries with chronic end-stage heart failure [17,18].

The Centers for Medicare & Medicaid Services guidelines for LVAD-DT eligibility in patients with chronic end-stage heart failure (New York Heart Association class IV end-stage left ventricular failure for ≤ 90 days with a life expectancy of < 2 years) and are not candidates for heart transplantation, mandate that they must meet all of the following conditions:

(1) The patient's class IV heart failure symptoms have failed to respond to OMM, including dietary salt restriction, diuretics, digitalis, β-adrenergic receptor-blocking agents, and angiotensin-converting enzyme inhibitors (if tolerated) for at least 60 of the past 90 days.

(2) The patient has a left ventricular ejection fraction of less than 25%.

(3) The patient has demonstrated functional limitation, with a peak oxygen consumption of less than 12 mL/kg per minute or the patient has continued need for intravenously administered inotropic therapy because of symptomatic hypotension, decreasing renal function, or worsening pulmonary congestion.

(4) The patient has the appropriate body surface area or size (≥ 1.5 m^2^) to support implantation of the ventricular assist device [17-19].

Notably, these coverage criteria do not address or stipulate several factors (e.g., cachexia, right ventricular heart failure, pulmonary hypertension, concurrent end-organ disease, malignancy or psychosocial factors) that are critical to patient outcome and QOL. Nevertheless, considering these factors is important in order to avoid, in some cases, reaching an ethically complex end-point sometimes referred to as "destination nowhere." [20].

In the INTrEPID trial (Chronic Mechanical Circulatory Support for Inotrope-Dependent Heart Failure Patients Who Are Not Transplant Candidates) conducted between March 2000 and May 2003, the Novacor device (World Heart Corp, Oakland, California) was used in LVAD-DT patients [12]. The survival rates for LVAD-DT (n = 37) and OMM (n = 18) were (46% vs 22%; *P *= .03) at 6 months and (27% vs 11%; *P *= .02) at 12 months.

In the post-REMATCH study conducted between November 2001 and December 2005, the in-hospital mortality rate was 27% for 280 patients who underwent HeartMate XVE implantation as DT [13]. The causes of death were infections with sepsis, right ventricular heart failure, and multiorgan failure. The overall 1-year, 2-year and 3-year survival rates were 56%, 31% and 17%, respectively. The predictors of poor 1-year survival were cachexia, poor nutrition, hematologic abnormalities, end-organ or right ventricular dysfunction, and lack of inotropic support in LVAD-DT. Stratification of LVAD-DT patients as having low (n = 65), medium (n = 111), high (n = 28), or very high (n = 18) risk on the basis of these predictors corresponded with 1-year survival rates of 81%, 62%, 28%, and 11%, respectively [13].

The post-REMATCH study used a case-series design; therefore, it did not validate the survival advantage attributed to LVAD in the subgroup analysis because it did not compare 1-year survival with comparable low-risk or medium-risk groups on OMM. Nonetheless, the excellent survival rate attributed to LVAD-DT in the low-risk subgroup can be similar to the survival rate with OMM in a similar group of low-risk patients (i.e., clinical equipoise). There has been an improvement in the overall survival rate after the onset of heart failure of 12% per decade during the past 5 decades [21]. The largest increase in survival is most noticeable in low-risk heart failure patients because of clinically significant advances in the development and utilization of multiple-drug regimens for OMM. Currently, the actual 3-year survival rate of risk-adjusted heart failure patients is 71% on OMM [22] and compares favorably with the survival rate of carefully selected low risk patients after LVAD implantation [14].

Advances in medical technology have enabled rapid and temporary application of short-term mechanical circulatory devices in emergency situations. Short-term mechanical circulatory devices include, but are not limited to, extracorporeal membrane oxygenation, percutaneous cardiopulmonary bypass or percutaneous ventricular assist devices. The short-term mechanical circulatory devices are initiated as a bridge to long-term (also called bridge to bridge) or a bridge to permanent surgical implantation of LVAD [16,23]. Idelchik et al. reported a series of 18 patients with chronic end-stage heart failure who experienced terminal hemodynamic collapse and underwent emergency placement of a percutaneous ventricular assist device (pVAD) (TandemHeart CardiacAssist Inc, Pittsburgh, Pennsylvania) as a bridge to long-term or permanent surgical implantation of an LVAD [16]. In Idelchik et al. study, patients survived pVAD and were successfully bridged to a permanent LVAD: 4 patients received a HeartMate XVE, 6 received a HeartMate II (Thoratec Corporation), 3 received a Jarvik 2000 (Jarvik Heart, Inc, New York, New York), and 2 received a Novacor device. The rate of survival with LVAD-DT was 73% at 30 days and 67% at 6 months. Gregoric et al. also reported emergent placement of pVAD in a series of 9 end-stage heart-failure patients [23]. The pVAD was a bridge to permanent surgical implantation of HeartMate II devices in 6 patients.

## Neurologic complications

The use of LVAD-DT is associated with neurologic complications, such as stroke, transient ischemic attack, toxic-metabolic encephalopathy, and cognitive dysfunction. A stroke is a devastating complication for the LVAD patient. It may be caused by an embolism from the device or a hemorrhage into the brain parenchyma because of coagulation disorders. The risk of stroke increases with the length of time on LVAD support. The time on LVAD before the occurrence of stroke can range from 30 to 500 days after implantation [11]. In the REMATCH trial, major neurologic complications occurred at a high rate in 30 (44%) of the 68 LVAD-DT patients with the HeartMate XVE compared with 4 (7%) of the 61 OMM patients [24]. In the INTrEPID trial, major neurologic complications occurred at a high rate in 23 (62%) of the 37 LVAD-DT patients with the Novacor device compared with 2 (11%) of the 18 OMM patients [12]. Strokes accounted for 34% of all deaths in LVAD-DT patients.

Of 124 patients with different ventricular assist devices implanted at the University of Pittsburgh Medical Center, 31 (25%) experienced strokes, with a mean time on ventricular assist device support of 228 days [25]. Of all strokes, 66% occurred within 4 months after LVAD implantation. Actuarial freedom from strokes at 6 months was 75%, 64%, 63%, and 33% with the HeartMate device (Thoratec Corporation), the Thoratec biventricular ventricular assist device (Thoratec Corporation), the Thoratec LVAD (Thoratec Corporation), and the Novacor device, respectively. The type of implanted device influenced the risk of stroke in LVAD. The risk of strokes in all patients was increased by the occurrence of antecedent infections irrespective of the device type. Long-term support with these devices can cause a nonfocal decline of global cognitive functioning in patients without a history of stroke, which is possibly related to brain damage in the frontal lobes [26].

## Infections and sepsis

**Figure 2:**
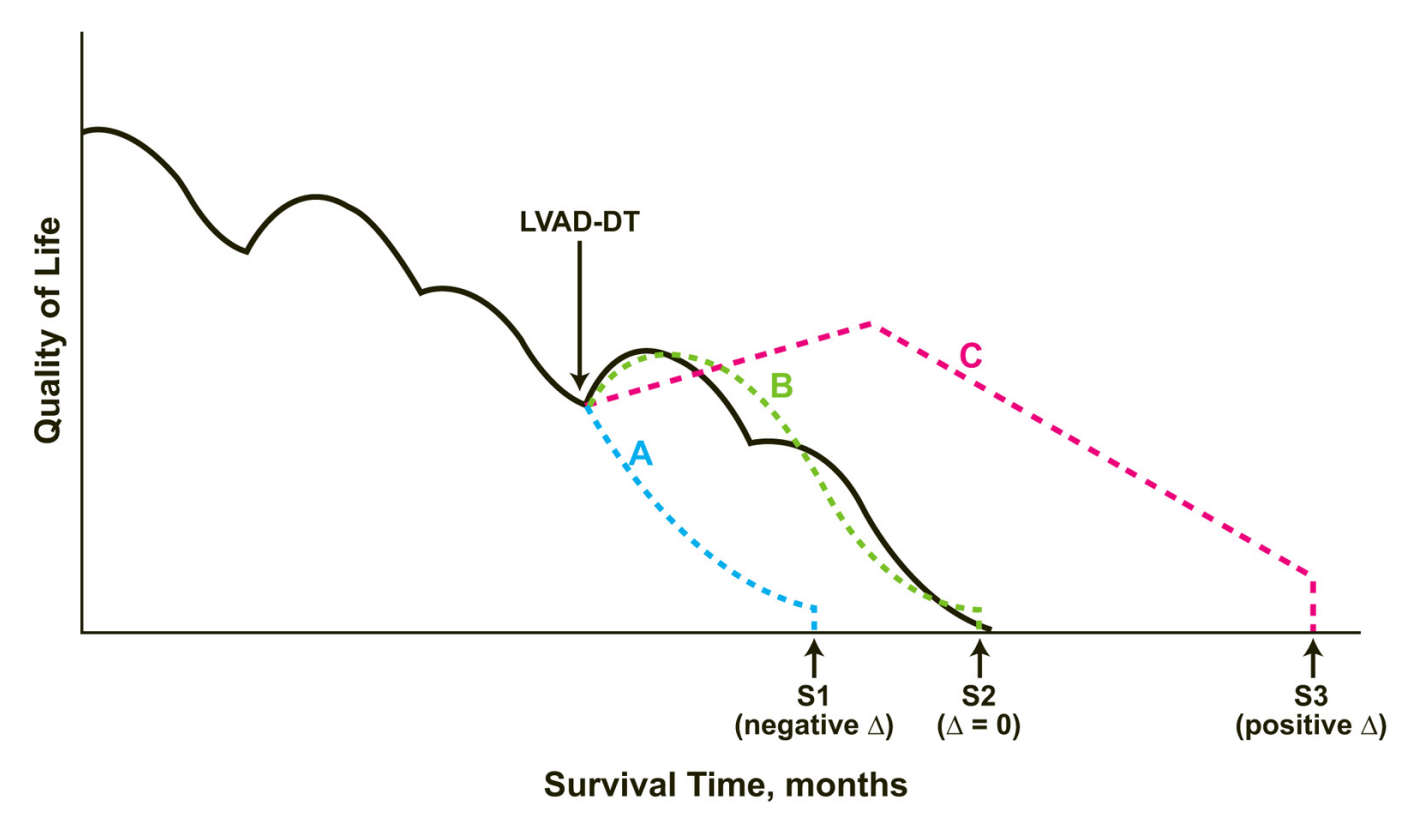
**End-of-life trajectory and quality-of-life (QOL) adjusted survival with left ventricular assist device (LVAD) as a destination therapy (DT)**. The effect of the use of an LVAD as DT (LVAD-DT) on quality of life (QOL) and survival in Medicare beneficiaries with chronic end-stage heart failure is evaluated by examining cumulative effects on multiple QOL domains (e.g., related to physical, mental, emotional, social, and financial areas), as well as the burden of disease and therapy on patients, caregivers, and family members. LVAD-DT had three possible effects (dotted lines) on QOL and end-of-life trajectory, compared with those of medically treated patients (solid line): (A) Premature decline in QOL with shortened survival time because of postoperative complications and high in-hospital mortality rate (range 14%–27%) within 90 days after device implantation [11-13,15,16]. In this situation, survival time (S1) is shortened by several months compared with survival in the medically treated patient. The LVAD is electively inactivated at the end of life resulting in abrupt death. (B) No substantial change in QOL or survival time (S2) compared with that of the medically treated patient. The LVAD is electively inactivated at the end of life resulting in abrupt death. (C) The LVAD-DT alleviates limitations of physical functioning related to left heart failure, therefore explaining an initial enhancement of QOL. A gradual decline in QOL appears over a lengthened survival time (S3) because of high combined rates of late serious complications such as infections, sepsis, neurologic disabilities, and device malfunction or failure beyond 90 days of device implantation. The progression of comorbid conditions such as pulmonary hypertension, extra-cardiac end-organ disease and active malignancy also exacerbate terminal decline in the overall QOL. In this situation, average patient survival time after LVAD-DT can be lengthened by about 12 to 24 months, compared with that of the medically treated patient [11-13]. The LVAD is electively inactivated at the end of life resulting in abrupt death.

Serious infections and sepsis are the most common complications after LVAD implantation and can occur in 18% to 59% of patients [27]. In the REMATCH trial, freedom from infections and sepsis in patients after implantation with LVAD-DT was 58% at 1 year and 48% at 2 years [28]. Infections can involve any part of the device and other organ sites, which increases the morbidity and mortality in these patients. Infections and sepsis accounted for 31% of all deaths in LVAD-DT patients over a 2-year interval in the REMATCH trial [11]. The onset of infectious complications and sepsis in LVAD-DT decreased the 1-year and 2-year rates of survival to 39% and 8%, respectively. In the INTrEPID trial, infections accounted for 24% of deaths in patients with LVAD-DT [12]. Infections also appeared to amplify the risk of acute strokes and permanent neurologic events in patients with ventricular assist devices [25]. It can be inferred from the published reports of clinical studies that LVAD-DT can add 12 to 24 months of survival time to an average Medicare recipient, with an estimated risk of 50% incidence of either serious neurologic or infectious complications during that time (Figure 2). These complications have consequences on QOL-adjusted survival and end-of-life trajectory for the average Medicare recipient.

## Device failure and replacement

Device malfunction or failure is defined as the inability of the device system to maintain adequate circulatory support [11]. Most device malfunctions or failures are life threatening but do not necessarily result in death. Device replacement may be required in some patients. In the REMATCH trial, 11 (16%) of the 68 LVAD-DT patients died because of device failure [29]. Freedom from device replacement was 87% at 1 year and 37% at 2 years [29]. In a retrospective analysis of 46 patients who underwent implantation with the HeartMate XVE (between July 2003, and March 2006), 13 of 15 patients had the device either removed or replaced by ≥ 330 days postimplantation to avoid unexpected mechanical failures [30]. LVAD-DT patients who experienced pump malfunction and subsequently required replacement of their HeartMate XVE (a first-generation pulsatile-flow pump) with HeartMate II (a second-generation axial continuous-flow pump) had increased morbidity and mortality after the surgical intervention [31]. Pathologic and histologic examinations of explanted HeartMate XVE (Figure 1) and Novacor devices from patients reveal significant degradation of structural integrity of bioprosthetic valves in the inflow and outflow conduits as early as 3 month postimplantation [32]. The bioprostheses valves in the inflow and outflow conduits are critical for long-term durability of HeartMate XVE and Novacor devices for DT. These bioprostheses valves are exposed to the immune system and hemodynamic forces soon after implantation. Over time, chronic inflammation along with mechanical forces weaken the valve cusps, leading to tissue degeneration with cusp tears, hemorrhage, and thrombus deposition. These pathologic changes can also explain the propensity of these devices to increase the combined risk of neurologic and infectious complications in implanted patients.

The HeartMate II device is implanted as a replacement of the HeartMate XVE in an attempt to decrease the risk of neurologic and infectious complications as well as device malfunction or failure in LVAD-DT [31]. Nonetheless, the continuous-flow LVAD-DT can accelerate the onset of right heart pressure overload and heart failure in patients with advanced heart failure [33]. The incidence of right heart failure after LVAD implantation can be as high as 35%, and it decreases the 180-day survival rate of implanted patients from 90% to 66% [34]. Continuous-flow LVAD-DT abnormally augments diastolic blood flow and pressure at the systemic capillary and arteriolar levels in end organs such as the brain, kidneys, and gastrointestinal tract during all phases of the cardiac cycle [33]. The physiological effects of hyperperfusion that augment diastolic blood flow and pressure can produce paradoxical vascular responses and end-organ injury. Stasis of arterial blood flow distal to partial atherosclerotic obstruction may increase the possibility of small-vessel occlusion. Small-vessel occlusion in the brain can manifest with neurological complications such as global cognitive decline and stroke. The development of arteriovenous malformations is another serious complication of dampened pulsatility and augmented diastolic blood flow and pressure by continuous-flow LVAD-DT. LVAD-DT patients can present with chronic gastrointestinal bleeding, which may resolve only after device explantation and orthotopic cardiac transplantation [35]. Therefore, in predicting end of life for patients with LVAD, it is equally important to determine the appropriateness of additional surgical intervention and the timing for replacement of a failed component or of the whole device because of serious malfunction.

## Quality of life

QOL domains include physical, psychological, social, spiritual, and financial well-being. The Short-Form 36 Health Survey (SF-36) consists of 36 questions that measure physical and mental aspects of the patient's QOL [36]. The SF-36 evaluates the patient's own perspective of physical functioning, bodily pain, role limitations due to physical health problems, general health vitality, social functioning, role limitations due to personal or emotional problems, and mental health.

In the REMATCH trial, LVAD-DT patients had higher scores than OMM patients in physical functioning (mean score, 46 vs 21) and in emotional functioning (mean score, 64 vs17) at 1-year follow-up [11]. However, the SF-36 was completed by small subsets of survivors in the LVAD-DT (n = 23) and OMM groups (n = 6) [11]. Therapy with an LVAD is expected to alleviate symptoms and limitations of physical functioning attributed to left heart failure. Nevertheless, the REMATCH trial did not address the effect of LVAD-DT on other SF-36 scores (e.g., bodily pain, social functioning, vitality, and mental health) and thus did not provide a comprehensive evaluation of QOL in survivors [11]. LVAD implantation can impose certain restrictions on social functioning. The potential of device failure requires patients to live close to a health care facility with the requisite expertise in LVAD technology [11]. Although patients can travel with the approval of their physician, doing so can be hazardous because of the potential development of emergency complications while traveling.

In the REMATCH trial, the Beck Depression Inventory (BDI) was used to measure the severity of depression in patients [11]. BDI scores range from 0 (normal) to 64 (severely depressed). At 1-year follow-up, the mean BDI scores were 8 and 13 for LVAD-DT (n = 23) and OMM (n = 6) patients, respectively. The difference in BDI scores between 8 and 13 on a 64-point scale is not clinically significant, because neither score indicates severe depression in either the LVAD-DT or the OMM survivors. Patients with an LVAD in situ had a higher incidence of anxiety disorders and poorer psychological functioning than did patients who had undergone transplantation or explantation of the devices [37].

Measuring the SF-36 or BDI scores before the occurrence of major neurologic complications, infections, device malfunction, or readmission to the hospital may not be meaningful nor may it ascertain the real QOL in an LVAD-DT patient. The patient's ability to perform activities of daily living and self-care can be severely compromised after a stroke, serious infection, or recent readmission to the hospital. An LVAD can have considerable effects on a patient's sense of self and perception of body image resulting in profound psychological sequelae for some patients and their families [38]. It is not uncommon that psychiatric symptoms, such as depression, anxiety, and posttraumatic stress disorders, are underdiagnosed and may be undertreated in patients with LVAD-DT, which can affect their overall QOL and survival [39].

Most patients with LVAD-DT died within 2 years of enrollment in the REMATCH, INTrEPID, and post-REMATCH clinical studies [11-13]. None of these studies provided measures of the quality of end-of-life care or palliation in the decedents in these clinical studies, which is surprising given the high incidence of complications before death. The physical health, levels of depression or anxiety, and the rate of posttraumatic stress disorders in caregivers were not assessed or reported in the LVAD-DT clinical studies. Caregivers included anyone who provided informal and nonpaid care to the patients at home (e.g., spouses, adult children or grandchildren, family relatives, significant others, or close friends). The quality of end-of-life care and dying, as well as the quality of caregivers' well-being, are considered important indicators of delivering patient-centered care and family satisfaction [40,41].

## Requirements of informed consent for LVAD-DT

The legal and ethical foundation for informed consent is: (1) the protection of patient autonomy and (2) the promotion of informed rational medical care decisions. The minimum requirements for informed consent include: (1) a description of the proposed treatment, its risks, and its benefits; (2) a description of alternate treatment options, including their risks and benefits; and (3) the voluntary granting of consent by a competent patient who understands the information presented. If a patient has impaired decision-making capacity, a legally designated surrogate should substitute in the informed-consent process [42].

The decision to undergo medical or surgical treatments is expected to be based on an informed patient choice [43]. On the other hand, it has been reported that patients undergoing invasive cardiac surgical procedures have a poor understanding of their disease, their intervention, and its complications making the attaining of true informed consent difficult, despite their desire to be informed of all risks [44]. LVAD-DT is a surgical procedure with significant consequences for patients' end-of-life care and trajectories (Figure 2) and with profound effects on caregivers and their families. Therefore, informed consent for LVAD-DT should include much detailed discussion and evaluation of particular elements before surgical implantation of the device (Table 1). It is essential to conduct a balanced discussion of medical management, palliation, and hospice care options, as well as to discuss the surgical procedure itself. Patients, caregivers, and families must become familiar with LVAD-DT complications, the progression of concurrent comorbid conditions, and the development of new diseases that can alter end-of-life trajectories (Figure 2). The possibility of survival with serious neurologic disability and a high burden of care require detailed explanation. Device-related complications can affect both the estimated recovery time before returning home, and the frequency of regular follow-up visits or unplanned readmissions for inpatient hospital care. This information can be presented by using various communication tools to ensure that patients, caregivers, and family members fully understand and appreciate the consequences of device implantation (Table 1). Surgical implantation should not be initiated until the clinician is convinced that the patient fully understands the risks, benefits, and reasons for the decision. Clarity and transparency in the consent process can help prevent subsequent major conflicts in end-of-life decision after implantation that require intervention by an institutional ethics committee [45].

**Table 1:** Requirements for informed consent for left ventricular assist device as destination therapy

**Participants**
• Patient
• Surrogate decision maker or medical power of attorney
• Caregiver (spouse, adult child, significant other, etc.)
• Primary care physician
• Palliative care specialist
• Cardiology specialist
• Cardiovascular surgical specialist
• Clergy
• Social services

**Information content**

• Description of end-stage heart failure disease and natural history
• Description of optimal medical management
• Description of palliative care and symptom management
• Description of hospice services
• Description of surgical procedure for device implantation
• Description of benefits from device implantation over optimal medical management
• Description of complications after device implantation
‣ Short-term operative death or complications
‣ Expected time of hospitalization and recovery from the surgical procedure
‣ Expected survival time after device implantation
‣ Expected quality of life (e.g., physical, psychological, social, and financial)
‣ Long-term complications
○ Device-related complications
▪Complications after open-heart surgery
▪Neurologic complications
▪Infections
▪Device troubleshooting
▪Device malfunction and failure
▪Pain
▪Noise and sleep-related disorder
○ Concurrent or new clinically significant comorbid conditions and diseases
‣ Notification or training of local hospital personnel and doctors
‣ Transfer to regional hospitals for inpatient specialized medical care
‣ Frequency of regular follow-up visits
‣ Frequency of inpatient readmissions
‣ Development of new intractable symptoms from right heart failure
‣ Advance care planning and documentation (e.g., in event of a stroke, serious infection, device replacement, loss of decision-making capacity, or disseminated malignancy)
‣ Anticipated end-of-life trajectories
‣ Palliative and hospice care with or without device implantation
‣ Device deactivation and death planning (when and where to "turn off" the device)
‣ Device procedures in end-of-life organ donation
• Description of short-term and long-term medical care costs with or without device implantation
• Caregiver burden after device implantation
‣ Physical
‣ Psychological
‣ Social
‣ Cultural
‣ Financial
‣ Daily life activities and work or employment

**Communication tools**

• Face-to-face interviews
• Device patients' support groups
• Caregivers' support groups
• Audiovisual media
• Electronic media (Web sites)
• Printed media (brochures)

**Verification of understanding of the relevant information (i.e., the patient and the caregiver should paraphrase the disclosed information)**

• Nature of the patient's medical condition
• Nature and purpose of the surgical procedure for device implantation
• Benefits and risks of device implantation
• Benefits and risks of optimal medical management
• Caregiver burden
• Anticipated changes in end-of-life trajectories
• Palliative and hospice care access with or without device implantation
• End-of-life care planning
• Mode of dying and death

**Validation of first-person (patient) informed decision making**

• Patient acknowledges seriousness of medical condition and likely consequences
• Patient compares medical and device therapies and consequences of each option
• Patient offers reasons for not selecting medical therapy as an option
• Patient offers reasons for selecting device implantation as an option

### Scope of palliative care in LVAD-DT

The World Health Organization encourages the use of palliative care in older persons with serious chronic progressive diseases such as chronic end-stage heart failure [46]. The World Health Organization defines palliative care as "an approach that improves the quality of life of patients and their families facing the problems associated with life-threatening illness, through the prevention and relief of suffering by means of early identification and impeccable assessment and treatment of pain and other problems, physical, psychosocial and spiritual" [47]. The World Health Organization palliative care criterion of neither hastening nor postponing death prevents the categorization of LVAD-DT as a form of palliative care. However, this criterion does not preclude providing palliative care in conjunction with life-prolonging therapy such as LVAD-DT. Palliative care focuses on managing intractable symptoms, preserving QOL, and communicating effectively with patients, caregivers, and families on end-of-life concerns. When palliative-care teams support the physical, emotional, and spiritual well-being of patients, caregivers, and their families, there is greater satisfaction with the overall quality of care [40,48]. The burden of unrelieved generalized pain, physical symptoms, and psychological symptoms has a negative impact on the QOL of patients with chronic end-stage heart failure [49]. Most of these patients express meaningful preferences about improving the quality versus the length of life [50]. Palliative care within a multidisciplinary team approach enhances the quality of end-of-life and shared decision making in chronic end-stage heart failure [40,48,51].

Palliative care can play an important role in conjunction with LVAD-DT in chronic end-stage heart failure (Table 2). LVAD-DT patients are often 65 years or older and may have clinically significant comorbid conditions. The early involvement of palliative-care specialists in the consent process for LVAD-DT is highly recommended because of the extremely invasive nature of LVAD-DT, the subsequent limitations on QOL-adjusted survival, and postimplantation alterations in end-of-life trajectories (Figure 2). Clinicians in medical and surgical specialties involved with the implantation and management of LVAD-DT may not be familiar with the full spectrum of available palliative-care options and the benefits (Table 2). Clinicians experienced in palliative care can best present the palliative-care options to patients, caregivers, and family members [52]. Therefore, the patient care team should include palliative care specialists early in the decision-making process well before implantation of LVAD-DT.

**Table 2:** Benefits of early palliative care in candidates considered for left ventricular assist device (LVAD) as destination therapy (DT).

• Apply early in the course of illness, in conjunction with other life-prolonging therapies to help patient better understand and manage new distressing symptoms and clinical complications
• Provide relief from pain and other distressing symptoms
• Enhance patient's quality of life
• Integrate the psychological and spiritual aspects of patient care
• Offer a support system to help patients live as actively as possible until death
• Offer a support system to help caregivers and family members cope during the patient's illness
• Affirm life, and regard dying as a normal process
• Intend neither to hasten nor postpone death
• Offer a support system to help caregivers and families with the grief reaction, including bereavement counseling after device deactivation and death

If a patient chooses to proceed with LVAD-DT after an informed consent discussion, palliative care specialists can continue to provide medical care within the multidisciplinary team approach. Continuity of palliative care is essential for the support of patients, caregivers, and family members and for their overall satisfaction with quality of medical care [40]. Palliative care specialists can intervene and counsel during follow-up as new physical, psychological and social problems develop in LVAD-DT patients. Furthermore, patients who initially agree to LVAD-DT may eventually decide to discontinue it. Patients may ask that such therapy be withdrawn in the event of a debilitating stroke, a severe infection, incapacitation by a comorbid condition, or the need for a second major surgery to replace device components [45]. Advance-care planning with palliative care specialists can smooth the transition to elective deactivation or the "turning off" of the device to allow progression to death [53].

### Caregiver burden

An important dimension of the informed-consent process is the effect of LVAD-DT on caregivers. A portable LVAD facilitates the discharge of patients from the hospital. However, discharging patients to go home can also increase physical, psychological, and financial strains on caregivers [54]. Caregivers need high levels of vigilance and education about these devices to detect early complications and troubleshoot device malfunctions [11]. Caregivers tend to worry more about device-related problems (e.g., malfunctioning, pain, infection, and stroke) than do the patients themselves [55]. Most of device malfunctions or failures that occur at home are frightening to caregivers. Caregivers are informally recruited to provide continuous care at home and to accompany patients to regular visits or unplanned hospital readmissions.

The intensity of long-term care required in different comorbid conditions determines the severity of physical and psychological consequences on caregivers [56]. The use of temporary or permanent, mechanical, life-support devices has profound physical and psychological consequences on caregivers. Mechanical ventilators are much more common than mechanical circulatory devices in clinical practice. However, there appear to be similarities in the long-term psychological and physical consequences for caregivers from the prolonged use of mechanical ventilators and mechanical circulatory devices. For example, caregivers of survivors on mechanical ventilators experience severe depression, anxiety, and posttraumatic stress disorders lasting as long as 12 months [57]. These disorders are incapacitating and prevent more than 20% of caregivers from returning to their normal daily life activities or previous employment. Poor physical health may also develop in caregivers, which can exacerbate depression [56]. Mechanical circulatory devices used as bridge or destination therapy result in a similarly high incidence of depression, anxiety, and posttraumatic stress disorders in caregivers [55,58].

LVAD-DT recipients are typically older and are therefore more likely to have spouses as caregivers who have health concerns of their own. The intensity of care required for LVAD patients at home can exacerbate physical health problems in caregivers. Feeling overloaded with responsibilities often produces psychological distress in the caregiver that can manifest as severe depression and posttraumatic stress disorder. Younger caregivers who provide home care are also at risk of developing financial difficulties if they are unable to continue working. Thus, a discussion of the potential effects on the physical health and the psychological, social, and financial well-being of caregivers should be an integral part of the decision-making process in LVAD-DT.

### Device costs and financial burden

In the REMATCH trial, the average total hospital cost of surgical implantation of LVAD in hospital survivors and nonsurvivors was $159,271 and $315,015, respectively [59]. The average cost for each hospital readmission for an LVAD recipient was $105,326. Nonetheless, the post-REMATCH study reported a 40% decrease in the average total hospital cost of LVAD-DT implantation to $128,084 per recipient in a select 23 of the 280 patients from $210,187 per recipient in 52 of 68 patients in the REMATCH trial [60]. Single centers have reported a much higher average total hospital cost per recipient for LVAD implantation ($197,957) than for cardiac transplantation ($151,646) [61]. The average cost per quality-adjusted life-year was estimated at $312,551 in an economic evaluation of 6 studies (1 randomized control trial, 1 case series, and 4 case reports between 1995 and 2003) that reported improved survival and QOL with LVAD-DT in chronic end-stage heart failure [62]. One US study reported a cost per quality-adjusted life-year of $36,255 to $60,057 for LVAD-DT [63]. The wide discrepancy in reported cost-effectiveness requires further analyses undertaken alongside randomized controlled trials and use of OMM and second-generation and third-generation ventricular assist devices.

The costs of short-term and long-term medical care required after device implantation should be explained in detail to patients. Regular office visits, unplanned visits to emergency rooms or hospital readmissions because of complications, device malfunctions, or unrelated illness can increase the financial burden for patients and caregivers alike. Patients residing in rural communities may have to travel long distances for follow-up visits at specialized centers. These financial implications must be explored with patients, caregivers, and family members during the consent process.

## Presumed and surrogate consent in emergency situations

We have outlined the prerequisite conditions for a first-person (the patient) voluntary informed consent for LVAD-DT. Advances in medical technology have enabled rapid and temporary application of short-term mechanical circulatory devices such as pVAD in emergency situations as a bridge to permanent implantation of an LVAD [16,23]. The feasibility of this technology in clinical practice raises a new ethical question: should presumed or surrogate consent substitute for first-person consent in emergency situations to authorize the use of these temporary short-term mechanical circulatory devices in patients with chronic end-stage heart failure? It has been argued that only first-person consent should permit LVAD-DT in chronic end-stage heart failure [64]. Three reasons have been proposed to justify first-person consent over presumed or surrogate consent: (1) the respect for patient autonomy, (2) the availability of less-burdensome end-of-life therapies, and (3) the noncurative elective nature of LVAD-DT as an end-of-life therapy. Presumed consent is the type of consent most relevant to procedures or interventions performed in emergency situations. Presumed consent arises when there are good empiric grounds for claiming that patients would consent if they could respond when asked [65]. Ideally, the relevant data would come from past choices that an individual patient had made in similar circumstances. The presumed consent approach is not applicable for LVAD-DT, however, because there are too many contingent variables (e.g., age, overall health, religious convictions, and financial resources) that influence a reasonable person's decision about this type of treatment. A reasonable person might choose to refuse a noncurative and burdensome treatment for a fatal disease. By this definition, the initiation of a short-term mechanical circulatory device as a bridge to permanent implantation of an LVAD would also qualify for first-person consent. Compare the following 2 emergency decision-making positions:

(1) According to position "0" (P_0_), in the absence of first-person consent, clinicians should use only *those procedures *(e.g., temporary circulatory support with cardiac medications only) that are *reasonable *to use and are not reasonable to not use.

(2) According to position "1" (P_1_), in the absence of first-person consent, clinicians should use *all procedures *(e.g., temporary circulatory support with cardiac medications and short-term mechanical circulatory devices as a bridge to permanent implantation of an LVAD) that are reasonable to use, even if it is equally reasonable to not use them.

There are 3 ethical considerations to consider in comparing P_0 _and P_1_: (1) patient-centered moral reasons (i.e., the promotion of the patient's best interests), (2) justice (i.e., the minimum care owed to a person in society while ensuring equality in distribution), and (3) utility (i.e., the promotion of the greatest benefit to the greatest number of individuals while inflicting the least amount of harm).

### P_0 _and P_1 _and the best interests of the patient

Are P_0 _and P_1 _equal in terms of respect for the patient's best interests when the patient's preferences are not known? In the absence of a well-informed surrogate or an advance directive, the clinician is just as likely to contradict what the patient would choose by going ahead with a procedure that is *reasonable to not perform *as it would be by going ahead with a procedure that is *reasonable to perform*. This premise is especially true for patients who are candidates for LVAD-DT because the patients will likely be of advanced age and in refractory chronic end-stage heart failure with other clinically significant comorbid conditions that preclude eligibility for cardiac transplantation. It is ethically, medically, and legally reasonable to withhold artificial life-sustaining treatments in a patient with a progressive terminal disease so as to allow a peaceful death [66].

There is a difference between P_0 _and P_1 _from a patient-centered moral perspective when the patient's preferences are not known. If, in an emergency situation, the clinician chose P_1_, but the treatment turned out to not be what the patient wanted, then this decision could be reversed. The temporary short-term mechanical circulatory device could be turned off. However, if P_0 _was chosen, the end result would not be reversible. Therefore, it can be postulated that voluntary withdrawal of life-support treatment may be morally equivalent to the refusal to initially begin that treatment. However, it does not follow that the patient and his or her family after P_1 _would find it just as easy to deal with treatment withdrawal as they would with treatment refusal initially (P_0_). It may be psychologically impossible for most patients to go through the process of shutting off short-term mechanical circulatory devices or deactivating ventricular assist devices including an LVAD after the device has been initiated, even if they might have elected to not have the device in the first place had they been afforded the opportunity to make that decision [45,67,68]. Hence, the clinician risks putting the patient or surrogate in the difficult and distressing position of having to make a decision that they never wanted to have to make. This type of decision can also create substantial cultural and religious conflicts and guilt feelings for surrogates and families.

Organ donation can also influence the process of deactivating mechanical circulatory devices and ventricular assist devices [69,70]. The Uniform Anatomical Gift Act was revised in 2006 to mandate the evaluation of all patients for end-of-life organ donation before the withdrawal of life support systems such as mechanical circulatory devices [71]. After the initial deactivation of a mechanical circulatory device for a declaration of death, the device may be reactivated in a donor for artificial circulatory support in order to preserve organs for transplantation. Concurrently, medications may have to be administered to suppress reanimation when the mechanical circulatory device is reactivated in a donor [72]. The initiation of mechanical circulatory devices as an end-of-life treatment and their subsequent conversion for organ preservation and donation without first-person consent poses serious cultural, religious, and perhaps legal dilemmas for clinicians, surrogates, and families.

### P_0 _and P_1_, justice, and utility

In emergency situations in which the clinician has no idea of the patient's preferences, withholding short-term mechanical circulatory devices and LVAD-DT would not seem to violate any duty to the patient. The more resources required for a specific treatment, the less plausible it is for that treatment to constitute the basic care to which all persons are entitled in a fair and equitable distribution of health care resources. There is no reason to suggest that withholding mechanical circulatory devices and LVAD-DT would constitute an injustice to patients who have not elected such treatment.

If P_0 _and P_1 _are equally just in LVAD-DT and neither leads to a substantial advantage for maximizing the good for a particular patient, then these positions will differ on utilitarian grounds. In general, P_0 _is clearly efficient. In contrast, P_1 _entails an asymmetrical use of resources without a proportional increase in benefits for the average patient. This discrepancy is the best reason for support of the need for first-person consent for LVAD-DT [64]. On a societal level, it is not cost-effective to use limited health care resources for a treatment whose benefits are comparable only to those resulting from not treating the patient, who might reasonably have chosen to forgo the treatment if given the choice [73].

Unlike P_1_, P_0 _eliminates potential financial reasons that might influence the decision-making process. Although at least one report suggests that LVAD therapy may cause a loss of revenue for health care facilities [61], participation in this new technology can have academic rewards and community prestige. Consistently applying P_0 _rather than P_1 _could also curb future research and development of short-term and long-term mechanical circulatory devices in clinical practice. It can be equally argued that surrogate consent should not replace first-person consent when short-term mechanical circulatory devices are used for temporary support in emergency situations as a bridge to permanent implantation of an LVAD. In an emergency situation, it may be difficult if not impossible to fulfill the prerequisite conditions outlined in Table 1 to ensure that surrogate consent is an informed process and in the patient's best interest.

## Conclusion

Compared with OMM, LVAD-DT can prolong the survival of recipients in chronic end-stage heart failure. Overall QOL can also be adversely affected in some recipients because of infections, serious neurologic disabilities, or device malfunction. LVAD-DT alters end-of-life trajectories. Caregivers can experience physical, psychological, and financial burdens after this type of treatment. Therefore, we advocate early utilization of a palliative care approach and outline the following conditions for informed consent: (1) direct participation of a multidisciplinary care team that includes palliative care specialists, (2) a concise plan of care for anticipated device-related complications, (3) careful surveillance and counseling for caregiver burden, (4) advance care planning for anticipated end-of-life trajectories and timing of the device's deactivation, and (5) discussion about the long-term financial burden on patients, families, and caregivers. In the absence of first-person consent, presumed consent or surrogate consent should be used cautiously before initiating short-term mechanical circulatory devices in emergency situations as a bridge to permanent implantation of LVAD in chronic end-stage heart failure. Future clinical studies of LVAD-DT should include measures of recipients' quality of end-of-life care and caregivers' burden.

## List of Abbreviations

BDI: Beck Depression Inventory; DT: Destination therapy; INTrEPID: Chronic Mechanical Circulatory Support for Inotrope-Dependent Heart Failure Patients Who Are Not Transplant Candidates; LVAD: Left ventricular assist device(s); LVAD-DT: Left ventricular assist device as destination therapy; OMM: Optimal medical management; P_0_: Position 0: in the absence of first-person consent, clinicians should use only those procedures (e.g., temporary circulatory support with cardiac medications only) that are reasonable to use and are not reasonable to not use; P_1_: Position 1: in the absence of first-person consent, clinicians should use all procedures (e.g., temporary circulatory support with cardiac medications and short-term mechanical circulatory devices as a bridge to permanent implantation of an LVAD) that are reasonable to use, even if it is equally reasonable to not use them; PTSD: Posttraumatic stress disorder; QOL: Quality of life; REMATCH: Randomized Evaluation of Mechanical Assistance for the Treatment of Congestive Heart Failure; SF-36: Short-Form 36 Health Survey; US: United States.

## Competing interests

The authors have no affiliations or financial involvement with any organization or entity with a direct financial interest in the subject matter or materials discussed in this manuscript. The authors declare that they have no competing interests.

## Authors' contributions

AGR, JLV, MYR and JLM attest that they have each made substantial contributions in drafting the manuscript and revising it critically for important intellectual content, that they have given final approval of the version to be published, and that they have participated sufficiently in the work to take public responsibility for appropriate portions of the content. AGR, JLV, MYR and JLM have each read and approved the final manuscript.

## Funding

The authors have no funding support to disclose.

## Supplementary Material

Additional file 1 Videos of HeartMate XVE and Thoratec ventricular assist devices implantation procedures. Click here for file
