# Supplementary material for: Ethical challenges with the left ventricular assist device as a destination therapy
Source: Philos Ethics Humanit Med. 2008 Aug 11;3:20. doi: 10.1186/1747-5341-3-20 (PMC2527574; doi:10.1186/1747-5341-3-20)
Supplement: Additional file 1 — Videos of HeartMate XVE and Thoratec ventricular assist devices implantation procedures. [file 1747-5341-3-20-S1.doc]

**Videos of HeartMate XVE and Thoratec ventricular assist devices implantation procedures.**

Ventricular Assist Device- How it Works (1 minute 23 seconds)

[http://www.youtube.com/watch?v=nI6RYN0ja6Y](http://www.youtube.com/watch?v=nI6RYN0ja6Y )

Heart mate 2 (4 minutes 28 seconds): Screening for Implantation and Patient selection

[http://www.youtube.com/watch?v=obukBK6jkRw](http://www.youtube.com/watch?v=obukBK6jkRw&amp;feature=related)

Heart mate 3 (10 minutes 22 seconds): Device Assembly and Set-up

[http://www.youtube.com/watch?v=tVu0rt3Jd6I](http://www.youtube.com/watch?v=tVu0rt3Jd6I )

Heart mate 4 (7 minutes 29 seconds): Patient Preparation and Implantation options

[http://www.youtube.com/watch?v=aDSrwCgdKvg](http://www.youtube.com/watch?v=aDSrwCgdKvg &amp;feature=related)

Heart mate 5 (6 minutes and 47 seconds): Outflow Graft Anastomosis and Percutaneous Tube Exit Sit Selection

[http://www.youtube.com/watch?v=bPKalAePa-g](http://www.youtube.com/watch?v=bPKalAePa-g  )

Heart mate 6 (4 minutes 46 seconds): Preparing the Ventricular Apex Conduit

[http://www.youtube.com/watch?v=4KMLjyTNm3E](http://www.youtube.com/watch?v=4KMLjyTNm3E  &amp;feature=related)

Heart mate 7 (6 minutes 24 seconds): Attaching the Outflow Graft to the Pump and Deairing

[http://www.youtube.com/watch?v=yfnBgMB5Abk](http://www.youtube.com/watch?v=yfnBgMB5Abk  )

Heart mate 8 (2 minutes 28 seconds): Electric Actuation of the XVE LVAD

[http://www.youtube.com/watch?v=gSc6j2OwXNU](http://www.youtube.com/watch?v=gSc6j2OwXNU   )

Heart mate 9 (1 minute 50 seconds): Anchoring the Pump

[http://www.youtube.com/watch?v=YkdTuyu36rk](http://www.youtube.com/watch?v=YkdTuyu36rk   )

Thoratec (2 minutes 30 seconds) System Overview

[http://www.youtube.com/watch?v=fjyzwFQJLGo](http://www.youtube.com/watch?v=fjyzwFQJLGo )

Thoratec 2 (1 minute 54 seconds) Cannulation Approaches

[http://www.youtube.com/watch?v=pvsi_vewEAU](http://www.youtube.com/watch?v=pvsi_vewEAU&amp;feature=related)

Thoratec 3 (6 minutes 12 seconds) Console Calibration and Startup

<http://www.youtube.com/watch?v=K2dZF35tl5o>

Thoratec 4 (3 minutes 19 seconds) VAD Implantation- Surgical Procedures

[http://www.youtube.com/watch?v=1J_jIVcLhR4](http://www.youtube.com/watch?v=1J_jIVcLhR4&amp;feature=related)

Thoratec 5 (1 minute 6 seconds) Bypass Cannulation

[http://www.youtube.com/watch?v=Wi4vOPHwR3c](http://www.youtube.com/watch?v=Wi4vOPHwR3c&amp;feature=related)

Thoratec 6 (1 minute and 44 seconds) LVAD Site Selection

[http://www.youtube.com/watch?v=-vGRsWUQZVU](http://www.youtube.com/watch?v=-vGRsWUQZVU&amp;feature=related)

Thoratec 7 (5 minutes 28 seconds) Apical Cannulation

<http://www.youtube.com/watch?v=a0_XnilP6V0>

Thoratec 8 (2 minutes 43 seconds) Aortic Graft Placement and Anastomosis

[http://www.youtube.com/watch?v=wFBlxJjdglo](http://www.youtube.com/watch?v=wFBlxJjdglo&amp;feature=related)
